# International genomic evaluation methods for dairy cattle

**DOI:** 10.1186/1297-9686-42-7

**Published:** 2010-03-01

**Authors:** Paul M VanRaden, Peter G Sullivan

**Affiliations:** 1 Animal Improvement Programs Laboratory, USDA, Building 5 BARC-West, Beltsville, MD 20705-2350, USA; 2 Canadian Dairy Network, 660 Speedvale Ave. West, Suite 102, Guelph, Ontario N1K 1E5, Canada

## Abstract

**Background:**

Genomic evaluations are rapidly replacing traditional evaluation systems used for dairy cattle selection. Higher reliabilities from larger genotype files promote cooperation across country borders. Genomic information can be exchanged across countries using simple conversion equations, by modifying multi-trait across-country evaluation (MACE) to account for correlated residuals originating from the use of foreign evaluations, or by multi-trait analysis of genotypes for countries that use the same reference animals.

**Methods:**

Traditional MACE assumes independent residuals because each daughter is measured in only one country. Genomic MACE could account for residual correlations using daughter equivalents from genomic data as a fraction of the total in each country and proportions of bulls shared. MACE methods developed to combine separate within-country genomic evaluations were compared to direct, multi-country analysis of combined genotypes using simulated genomic and phenotypic data for 8,193 bulls in nine countries.

**Results:**

Reliabilities for young bulls were much higher for across-country than within-country genomic evaluations as measured by squared correlations of estimated with true breeding values. Gains in reliability from genomic MACE were similar to those of multi-trait evaluation of genotypes but required less computation. Sharing of reference genotypes among countries created large residual correlations, especially for young bulls, that are accounted for in genomic MACE.

**Conclusions:**

International genomic evaluations can be computed either by modifying MACE to account for residual correlations across countries or by multi-trait evaluation of combined genotype files. The gains in reliability justify the increased computation but require more cooperation than in previous breeding programs.

## Background

Today, selection in many countries uses genotypes in addition to phenotypes and pedigrees [1,2]. More than 50,000 dairy cattle worldwide have been genotyped for 50,000 markers. Breeders can select globally from the best animals if national evaluations with similar properties can be compared fairly and accurately. Changes from genetic to genomic evaluations for dairy cattle at the national level will require corresponding changes to international evaluations.

Phenotypes are collected, stored, and evaluated independently by each country, and the resulting estimated breeding value (EBV) files are exchanged and combined by Interbull. Multi-trait across-country evaluations (MACE) for nearly 30 traits are provided routinely using the methods developed by Schaeffer [3]. Results are distributed only for proven bulls with daughters in at least 10 herds. New methods are needed to exchange and combine genomic EBV (GEBV) files that include young bulls and perhaps also females.

National evaluations are deregressed to separate information from parents and progeny and provide a vector of observed phenotypes (**y**) within each country. These are combined by MACE in a weighted analysis. Statistical analyses of national evaluations are simpler after separating these sources of information by deregressing the prior information that already regressed the phenotypic deviations toward the parent average, and toward the population mean, or toward 0. Daughter yield deviations may be available even if the full data vector is not, or **y **may be approximated by backsolving from the traditional evaluations, using the reliabilities and the pedigree file (a list of each animal and its parents). Deregressed EBVs can be obtained using either sire-maternal grandsire [4] or sire-dam [5] pedigrees. Deregressed EBVs are recommended as the **y **variable in genomic evaluations [6]. Methods are developed here to deregress GEBVs for use as the **y **variable in international evaluations.

Genetic by environmental interactions can be predicted by genotyping each animal just once instead of obtaining phenotypes for each animal in each environment with traditional evaluation. High reliability requires very large data sets to estimate the small effects of individual genes [7]. Thus, breeders should consider combining or exchanging genomic data across countries to increase reliability. Advantages of international selection programs are large if genetic correlations among countries are high, if populations are genetically similar, and if markets for genetic material are already well established.

National evaluations often use linear models for normally distributed traits or nonlinear models for traits with non-normal distributions, but international evaluations are usually restricted to linear models for simpler computing. Examples are national threshold models for categorical traits such as calving ease that are then combined by the International Bull Evaluation Service (Interbull) using standard linear mixed models. Linear model equations for genomic selection were first developed by Nejati-Javaremi et al. [8] and are nearly as accurate as nonlinear equations for most traits [1].

The objectives of this paper are to 1) summarize methods for computing and deregressing national GEBVs, 2) compare methods for incorporating national EBVs and GEBVs into international GEBVs, and 3) illustrate benefits from exchanging GEBVs or exchanging genotypes.

## Methods

### Deregression of national evaluations

Traditional national EBVs () are often computed by animal model methods [9] and for a single trait (e.g. milk yield) can be represented approximately using a vector of daughter deviations (**y**), a diagonal matrix containing daughter equivalents (**D**), an additive relationship matrix (**A**), and a variance ratio (*k*) as:

Genomic EBVs () within each country can be represented approximately by replacing the pedigree relationships from **A **by the genomic relationship matrix (**G**), giving

Matrix **G **can be computed from genotypes as a quadratic form and can also include polygenic variation from **A **that is not linked to the markers [10]. Ratio *k *is a function of heritability (*h*^2^) and was defined as  by [9]derivation or as  by Fikse and Banos [11], with mate breeding values assumed known or unknown, respectively. Elements of **D**, known as daughter equivalents or effective daughter contributions, must match the definition of *k*.

For traditional MACE, elements of  and pedigree files are provided to Interbull, and elements of **y **are backsolved from these. In the simplest case, **y **could be obtained by pre-multiplying  by **D**^-1^(**D**+**A**^-1^*k*). However, vector  should contain solutions from all ancestors including unknown parent groups, but some are not included in the exchange formats, and the MACE model also includes an additional fixed effect of the country mean, all of which must be solved using either iterative or other methods. Elements of **y **equal 0 for the ancestors and group effects because these are not observed directly, and matrix **A**^-1 ^contains coefficients that link animals with observations to ancestors and unknown parent groups.

For genomic MACE (GMACE), diagonal matrix **D**_*g *_can contain the extra daughter equivalents from genomic data. Diagonals of **D**_*g *_can be calculated in at least three ways (**D**_*g*1_, **D**_*g*2_, and **D**_*g*3_). The first method calculates diagonals of **D**_*g*1 _from the difference between genomic reliability (*REL*_*g*_) and traditional reliability (*REL*) for each bull simply as

The second method obtains elements of **D**_*g*2 _by reversing standard reliability formulas like those of Misztal and Wiggans [12] such that the diagonals of the matrix (**D**+**D**_*g*2_+**A**^-1^*k*^-1^) equal or approximate the diagonals of (**D**+**G**^-1^k^-1^).

The third method is the simplest and sets all diagonals of **D**_*g*3 _equal to the same constant. When **G **becomes too large for inversion, this simple strategy will still be affordable. Traditional *REL *expressed as decimals rather than percentages are summed and reliabilities of the corresponding parent averages (*REL*_*pa*_) are subtracted for all genotyped animals. This result is multiplied by variance ratio *k *and divided by factor *n *to determine average daughter equivalents from genomic data. A value of *n *equal to 1500 for Holsteins, 1200 for Brown Swiss, and 700 for Jerseys is used to match estimated reliabilities to those observed from truncation studies in US breed evaluations [13]. An interpretation of *n *is the number of high reliability bulls needed to obtain 50% *REL*_*g*_, and a larger *n *is needed for breeds with greater effective population size [14].

Algebraically,

Equality of approximate and published genomic reliabilities is an advantage of the second method. If the first or third method is used in GMACE, *REL*_*g *_will be biased upwards for genotyped animals with many relatives because genomic information in **D**_*g *_is counted twice, once directly and once via relatives.

Matrix **G **is not expected to be available to Interbull for the Holstein breed, whereas vector  is available. In North American evaluations, **G **is already a 30,000 × 30,000 dense matrix and is rapidly growing larger. Let **y**_**g **_contain deregressed evaluations derived from the national , which includes both the traditional and the genomic information. Vector **y**_**g **_is obtained from  using equations

The equations are solved iteratively because elements of **y**_**g **_equal 0 for unknown parent groups whereas corresponding elements of  must be estimated. As was the case for national models, **D **and **D**_**g **_must now match the international definition [11] used for variance ratio *k*, which may or may not be the same definition that was used nationally [9]. Matrix **A**^-1^ distributes the genomic information in **y**_**g **_to close relatives in the same way that phenotypic information is distributed.

Genomic estimated breeding values (GEBV) can be decomposed into the parent average (*PA*), the deviation of traditional EBV from PA (estimated Mendelian sampling), and the deviation of GEBV from EBV (additional genomic information):

The total daughter equivalents (*DE*_*total*_) can be similarly partitioned into:

Furthermore, the extra daughter equivalents from genomics (*DE*_*gen*_) can contain daughter equivalents from foreign daughters used to estimate SNP effects that are not included in the domestic daughter count *DE*_*dau*_. The traditional reliability from domestic daughters (*REL*_*dau*_) is

Deregression uses matrix algebra, but can be represented approximately for bull j as division by *REL*_*dau *_to obtain the original daughter average before regression. The approximate formula *EBV *= (*REL*_*dau*_)*y*_*j *_+ (1-*REL*_*dau*_)*PA *can be rearranged to solve for *y*_*j *_as:

Variance of vector **y **is partitioned into additive relationship matrix **A **and diagonal matrix **D**^-1 ^containing variance of residuals:

Diagonals of **D**^-1 ^for each bull are  or equivalently .

### Exchange of genomic estimated breeding values

Traditional MACE combines information from domestic and foreign relatives to increase reliability. Information from daughters contributes directly to **D **and **y **whereas information from ancestors and sons contributes indirectly through **A**^-1^. MACE equations are very similar to those used for deregression with the following exceptions: diagonals and **y **from all countries are stored together in the same vector, genetic correlations across countries are accounted for using the Kronecker product of **A**^-1 ^with the genetic covariance matrix inverse (**T**^-1^), use of **T**^-1 ^instead of *k *requires dividing the diagonals of **D **by , and vector  includes an *EBV *for each bull on each country scale obtained using equations:

Genomic MACE includes genomic information by applying deregression to national GEBV instead of EBV to obtain elements of **D **+ **D**_**g **_and **y**_**g**_. Vectors and matrices are extended to include data from multiple countries, and vector  includes international GEBVs on each country scale obtained using equations

If any countries have used foreign data to estimate marker effects, then errors in **y**_**g **_are no longer independent and should be modelled using the more general matrix **R **instead of **D **+ **D**_**g**_. Approximate formulas to compute **R **are proposed in the next section.

### Correlations among national evaluations

Exchange of genomic data between countries introduces additional correlations among their national evaluations that need to be modelled in GMACE. Residual effects can be correlated with residuals in other countries for two reasons: 1) multiple evaluation centers may include genomic and phenotypic data from foreign animals in national estimates of marker effects, and 2) genomic predictions act as repeated measures of the same portion of genetic merit rather than independent measures of genetic merit, especially for major gene marker(s). As an example of 1), marker effects in Canada and the United States may be highly correlated because the countries share genomic data and include MACE evaluations as input to the genomic equations in each country. As an example of 2), multiple countries could each test a bull for DGAT1, a gene with major effects on milk yield and components [15], and these repeated tests in different countries would not provide independent information about the bull's total breeding value.

Residuals are independent in traditional MACE because each daughter is measured in only one country, but may be correlated in GMACE for the reasons described above. In genomic MACE, diagonals of **R **should be  and off-diagonals can be non-zero due to residual correlations that depend on the ratio  in each country. Correlations are nonzero when more than one country submits GEBV for the same genotyped bull. Let d_1 _and d_2 _be the ratios  in country 1 and country 2, respectively, and let c_12 _be the fraction of genotyped bulls in common. For countries that share all genotypes, c_12 _may be 1 whereas c_12 _may be close to 0 for country pairs that only include genotypes of domestic bulls. The correlation of residuals e_1 _and e_2 _may be approximated using the additive genetic correlation, the fraction of common bulls, and the proportions of genomic information as:

The genetic correlation corr(a_1_, a_2_) between true breeding values (BVs) in countries 1 and 2 is routinely estimated by Interbull and acts as an upper limit for the residual correlation corr(e_1_, e_2_) because marker effects differ in different environments, just as BVs differ. MACE equations may need just a few changes to accommodate GEBV. A bull's diagonal in country *i *(**R**_*ii*_) depends as above on  instead of only :

Off-diagonals for the same bull in country *i *and *j *(R_ij_) are obtained by multiplying corr(e_i_, e_j_) by , giving:

### Simulated genotypes

A world population was simulated and evaluated to test the ability of multi-country methods to combine information from genotypes or GEBV computed separately within each country. Genotypes and phenotypes were simulated using pedigrees and reliabilities for all 8,073 proven Brown Swiss bulls in the April 2009 Interbull file. Genotypes and true BV for another 120 young bulls born and sampled in the United States with no progeny records yet were simulated to test the predictions. Brown Swiss genotypes were simulated because Interbull is conducting research with actual genotypes for this breed.

Genotypes for 50,000 markers and 10,000 QTLs were simulated using the same methods as VanRaden [10]. Markers and QTL were in equilibrium in the earliest generation and transmitted to descendants with recombination from crossovers on 30 chromosome pairs. To make QTL effects correlated across countries, independent normal effects within each country were multiplied by the Cholesky decomposition of the genetic correlation matrix among countries. Then, QTL effects were transformed from standard, normal distribution (*z*) to heavy tailed distribution (*q*) using *q *= *z *(1.9)^(*abs*(*z*)-2) ^such that the largest *q *explained 1-4% of genetic variation. Genetic correlations in the simulation were set equal to official estimates from Interbull [16]. Official correlations differ from correlation estimates due to post-processing to ensure positive definiteness and averaged about 0.90 but were lower for New Zealand than for the other countries.

Phenotypes equalled true BVs plus an error with variance determined from each bull's *REL *for protein yield. The 10,000 QTL effects were summed to obtain true BV. Only one replicate was simulated to demonstrate the computations. For both proven and young bulls, observed reliabilities were computed as squared correlations of estimated with true BVs on all nine country scales.

### Actual genotypes

Actual genotypes for 10,129 Holstein bulls and cows that had either daughters or records for protein yield in North America were also used to test multi-country models. Of these Holsteins, 7,928 had information only in the United States, 1,730 only in Canada and 471 in both countries. Evaluations on both scales were also computed for 11,815 young bulls and heifers, for a total of 21,944 genotyped animals. Results for the 2-country US-Canada Holstein test are not presented because MACE rather than Canadian national EBV were used as input data. Thus, only timing and convergence tests are presented.

### Direct genomic evaluation

Countries that share common genotype files could model foreign evaluations as correlated traits by computing a direct multi-trait genomic evaluation. Instead of converting foreign evaluations to the domestic scale and then assuming that foreign and domestic information measures the same trait, deregressed EBVs from multiple countries can each remain on the original scales. Information is combined in a multi-trait evaluation using genomic rather than pedigree relationships and the published genetic correlations. GEBVs for each bull on each scale are obtained using

The analysis uses genotypes directly to form **G **but not phenotypes directly because deregressed national EBVs are the input data rather than raw phenotypes. Residuals are then independent for the **y **vector in this analysis. Matrix **G **is larger than in national evaluations because it includes genomic relationships among all bulls genotyped internationally.

### Tests performed

Five evaluation systems were applied to the simulated Brown Swiss data. The five models were 1) national evaluation using pedigrees and phenotypes within countries, 2) MACE using pedigrees and phenotypes across countries, 3) genomic evaluation using genotypes and phenotypes within countries, 4) genomic MACE using genetic correlations to combine the within-country GEBVs into across-country GEBVs, and 5) multi-trait genomic evaluation using genotypes and phenotypes across countries. For all five systems, the young bulls predicted were domestic on US scale but were foreign on all other scales, which would affect the observed reliabilities.

Evaluation system 5 was applied to the North American actual Holstein genotypes only to determine if the computation required was reasonable; gains in reliability were not tested. The deregression methods were also tested on actual US Holstein data, and the resulting daughter equivalents from genomics and deregressed EBVs were compared. The iterative, nonlinear program used to compute US official genomic evaluations required only a slight modification to compute a multi-country genomic evaluation. Inverses of genetic correlation matrices have large off-diagonals that are multiplied by the square root of the product of the variance ratios for each country pair in the mixed model equations. Convergence was nearly as fast for multi-country as for single-country analysis if a block-diagonal solver was used.

### Genomic reliability

Reliability of GMACE evaluations will also be affected by residual correlations. Genomic information increases reliability, but if genotypes are shared by some countries, "double-counting" of this shared information should be avoided. Methods to approximate reliability of GMACE evaluations and account for the residual correlations are being developed. A possibility is to use multi-country deregression to backsolve for independent **y **from each country so that the current formulas to compute MACE *REL *can also be used for GMACE *REL*_*g*_.

Reliabilities for direct multi-country GEBVs can be obtained by including genomic relationships in matrix inversion, but computing costs for multi-trait equations may be too large. Reliability increases with the number of genotyped animals that also have phenotypes. Reliabilities for GMACE can be approximated by accumulating information chronologically to ancestors then progeny [12,17], but by using multiple-trait rather than single-trait equations when accumulating information [18,19]. Software used currently to approximate reliabilities for regular MACE uses single-trait equations but could be modified for GMACE to use multiple-trait equations instead.

## Results

Deregression of national genomic evaluations was tested on the US Holstein data. Differences between calculated **D**_*g *_from the three methods were small in proportion to **D **for sires with many genotyped progeny because those sires also generally had many daughter records. For the genotyped bulls with daughters, mean diagonals of **D**_*g*1_and **D**_*g*2 _were 19.4 and 19.1, respectively, both with SD of 11.3, and a correlation of 0.992. However, for young bulls without daughters, the differences were slightly larger. Means of **D**_*g*1 _and **D**_*g*2 _were 23.5 and 22.9, respectively, with SD of only 1.2 and 1.4, and a correlation of 0.81. The very simple approximation **D**_*g*3 _does not account for the number of close relatives genotyped and instead assigned the same constant of 22.3 to all bulls. Any of the three methods could be useful because of their similar properties.

The deregressed GEBVs in vector **y**_**g **_were very similar when computed using the three different **D**_*g*_. Correlations exceeded 0.999 among each of these for both proven bulls and young bulls. Means and SD were also nearly identical, except that the SD was about 1% higher for young bulls in **y**_**g **_computed using **D**_*g*2 _instead of **D**_*g*1_or **D**_*g*3_. Results indicate that the choice of deregression methods might not affect GEBV but will affect computed *REL*_*g *_slightly.

### Exchange of genomic estimated breeding values

Young bulls tested in more than one country can have large residual correlations in GMACE, and these correlations need to be accounted for to prevent inflation of the resulting GEBV and reliabilities. Numerical values of corr(e_1_, e_2_) are shown in Table 1 for young bulls (those with *DE*_*dau *_= 0 in both countries) and for proven bulls (those with *DE*_*dau *_> 0 in at least 1 country).

**Table 1:** Residual correlations for country pairs with 0.90 genetic correlation and 100% genotype sharing (c_ij _= 1)

Daughter equivalents from progeny		Daughter equivalents from genomics		Residual correlation
	
Country 1	Country 2	Country 1	Country 2	
0	0	20	0	0.00
0	0	20	20	0.90
100	100	20	0	0.00
100	100	20	20	0.15
100	0	20	20	0.37
1000	1000	20	20	0.018

Tables 2 and 3 show observed reliability as measured by squared correlation of estimated and true BV for old and young bulls from the five evaluation systems tested. Countries are listed by population size in both tables, and traditional *REL *tend to be higher for large populations because more progeny are obtained per bull. Traditional national reliabilities for young bulls in Table 3 were the observed *REL*_*pa *_and were fairly low because the US bulls had no daughters in any country and may have had few close relatives in other countries. Also, information was contributed only by sires and maternal grandsires and not dams. Traditional MACE increased *REL*_*pa *_for the young bulls, but only a little. National genomic *REL*_*g *_were higher than traditional *REL *in the larger countries but not in the smaller countries, and were lower in some cases in Table 2 with very small numbers of proven bulls.

**Table 2:** Average reliability for proven bulls after exchanging traditional evaluations (MACE), genomic evaluations (GMACE) or genotypes

	Brown Swiss	Traditional		Genomic		
		
Country	Bulls	National	MACE	National	GMACE	Multi-country
Germany	4,414	81	82	84	84	84
Switzerland	2,184	90	91	91	91	92
Italy	1,390	87	89	88	89	89
United States	730	78	81	83	85	88
Slovenia	280	89	92	90	93	93
France	233	80	81	88	90	90
Canada	135	74	86	72	87	90
Netherlands	101	82	90	80	91	91
New Zealand	34	64	65	60	65	78

**Table 3:** Average reliability for young US bulls after exchanging international phenotypes (MACE), genomic evaluations (GMACE), or genotypes

	Traditional		Genomic		
	
Country	National	MACE	National	GMACE	Multi-country
Germany	4	11	64	68	69
Switzerland	14	17	65	70	73
Italy	1	12	34	60	64
United States	20	17	55	69	70
Slovenia	0	11	6	58	55
France	2	15	21	67	66
Canada	1	14	9	59	61
Netherlands	2	13	6	59	58
New Zealand	1	1	1	30	26

Application of GMACE to the simulated Brown Swiss data revealed large gains in *REL*_*g *_for young bulls. Gains from GMACE were small for old bulls because traditional *REL *was already high. In the GMACE evaluation, all countries had genotypes of young US bulls available, and computed the national GEBV for the same set of young bulls, but did not share the genotypes of reference bulls. This may not be realistic, but provided a simple test that the GMACE software can effectively combine genomic information across countries using GEBVs instead of genotypes. The time required for GMACE was less than 15 min on a single processor. Within-country genomic evaluations were required as inputs to GMACE, however the times required to compute these were much less than for multi-country evaluation because genotypes of foreign proven bulls were not included.

Actual correlations among GEBV from different countries should be documented as these become available. Ability of GMACE to model residual correlations could be tested with simulated Brown Swiss data, but application to real data is needed to reveal potential problems or refinements needed. Such studies are planned for the near future.

### Direct genomic evaluation

Observed reliabilities from direct, multi-trait evaluation of simulated genotypes in Tables 2 and 3 were similar to those from GMACE evaluation for both proven and young Brown Swiss bulls. All countries benefited from multi-country analysis. The countries with smaller populations such as Canada, Netherlands, and New Zealand had the largest gains in reliability for both young and old bulls. Countries with larger populations such as Germany and Switzerland also benefit and may gain the most by ensuring that their breed keeps pace with gains in other breeds instead of falling behind due to lack of cooperation.

Times required for 250 iterations were tested using two compilers. With the Absoft compiler and automatic parallel option (-apo), nine processors took 30 h for the 9-country Brown Swiss genomic evaluation and two processors took 11 h for the 2-country Holstein evaluation. With the Intel compiler, a single processor took 71 h for the Brown Swiss analysis and 6.5 h for the Holstein analysis. Total processor time increased linearly with number of countries with Absoft compiler but less than linearly with Intel. For both compilers, time required for iteration increases linearly with the number of bulls that have daughters. Time required for exact reliability calculation may increase dramatically, in proportion to the number of countries cubed, because dimensions of the matrix to invert are multiplied by the number of countries in the analysis. Matrix sizes might be reduced by including multiple equations only for the bulls with data in multiple countries rather than for all bulls. Approximate reliability formulas will be needed if inversion times are eight times larger with two countries than with one.

Correlations assumed in multi-country evaluation had very little effect on convergence rate but can have large effects on the direct genomic values (DGV), particularly on scales where large proportions of bulls are foreign and have converted information. Genetic group effects were not simulated and unknown parent groups were not included in the Brown Swiss test, but will be needed to account for selection in actual data.

## Discussion

### Comparison of evaluation systems

Reliability of selection for young animals greatly increased when national and international genomic evaluation models were applied to simulated data. Traditional MACE increased reliability for young animals by transferring pedigree information across countries. Genomic evaluations within country increased reliability, especially for countries with large populations. Multi-country evaluation of combined genotypes increased reliability further, especially for countries with small populations. Genomic MACE produced reliabilities almost equal to those from the combined genotype evaluation for the special case where the young bulls had GEBV on each country scale even though countries did not share genotypes of proven bulls. Thus, genomic information can be transferred by combining either GEBVs or genotypes.

Computing time was much faster for GMACE than the combined genotype evaluation. For GMACE, genomic predictions were computed using only the domestic proven bulls rather than all 8,073 proven bulls. Then, the within-country predictions were combined across countries in only 15 min using matrix **A**^-1 ^which is sparse whereas matrices **G **and **G**^-1 ^are dense. Thus, GMACE should be computationally feasible for the world Holstein population. Software for GMACE is in C rather than Fortran and was compiled with generic gnu compiler 'gcc'.

Future research should focus on including both genotyped and non-genotyped bulls in multi-country analyses, incorporating animal model pedigree for the non-genotyped bulls, accounting for dams' evaluations that may be biased, and perhaps including multiple traits per country. The approximations that account for correlated residuals among GEBV in GMACE need to be validated for applications involving many countries with different patterns of genotype sharing.

Marker effects may be highly correlated if countries share the same genomic data and include traditional MACE evaluations as input to their genomic equations. Countries could compute independent, less accurate GEBVs from only domestic data for exchange within Interbull, but such evaluations are not needed if the official GEBVs that contain both domestic and foreign data can be exchanged using genomic MACE.

Correlations caused by repeated tests of major genes are not specifically accounted for in this approximation. High-density chips such as 50,000 or 500,000 SNPs may not completely explain all the genetic variance because true QTL effects are between the markers. Partitioning the genetic variance into explained and unexplained components may require more complex models including polygenic effects.

### Implementation

To compute national GEBV, countries still need to receive conventional MACE EBV as input data for any foreign bulls whose genotypes they include. If MACE GEBV were used as input data, genomic information would be counted twice. The MACE programs revised as above could be used to evaluate both EBV and GEBV. The GEBV analysis simply reduces to the conventional MACE EBV if all countries supply EBV. The proposal is for all countries that report GEBV to also report EBV in a separate file and for Interbull to process and report both GEBV and EBV back to member countries. This can be achieved using the current formats, perhaps including a code to indicate which bulls have been genotyped.

Genomic selection will cause selection biases in conventional national evaluations. About three to four years after implementation, average Mendelian sampling will no longer equal 0 for bulls with progeny. To avoid EBV bias, simultaneous analysis of phenotypic, genomic, and pedigree data may be needed to properly account for selection on genotypes, rather than solving for EBV and then GEBV in a two-step process [20]. Countries may need to provide phenotypic summaries such as daughter yield deviation (DYD) instead of only GEBV to help users understand data sources.

Accurate blending of genomic and non-genomic information is important because many animals are not genotyped. Reliability can be improved directly by genotyping an animal or indirectly by genotyping close relatives. The extra information from genotyped parents can be transferred to non-genotyped descendants using the same formulas that adjust traditional evaluations for foreign parent data. Propagation from genotyped progeny to non-genotyped parents is more difficult because the extra information from genotyped progeny should not exceed the direct gain from genotyping the parent. Simultaneous evaluation of national phenotypic and genomic data such as proposed by Legarra et al. [20] could increase reliabilities for genotyped animals and for their non-genotyped ancestors and descendants.

Multi-trait, combined genotype evaluation required solving effects for more than one country scale together in the same program. Total computing time was nearly the same for combined as for separate country analyses. Instead of one computer doing US evaluations and another doing Canadian evaluations, two computers could each process half of the traits to complete the combined evaluation in the same time. The multi-trait genotype evaluation has the theoretical advantage that domestic proofs from both countries could be used directly instead of using domestic proofs from one country plus MACE proofs from the other.

The exact multi-country analysis of shared genotypes will be useful to judge properties of these approximations and can be implemented to increase reliability among sets of countries that do share genotypes. Use of different SNP chips by different organizations may make genotype sharing more difficult unless efficient methods to impute genotypes are found. A potential problem with genotype sharing is that countries or organizations that invest little in genotyping or phenotyping may benefit as much as those that invest more, which will reduce incentives to collect and provide additional data. The political decisions regarding genomics may be more important than the mathematical formulas and computer methods derived here.

## Conclusions

Genetic progress increases if national and international evaluations include genomic information. Previously, international evaluations did not include young bulls and females but at present, they should because of their increased reliability and because maximum progress requires shorter generation intervals. Methods were developed to combine GEBV files using GMACE or to compute multi-country evaluations if genotype files are shared. Advantages of GMACE are: similarity to the current MACE system, ability to account for residual correlations when countries include foreign phenotypes in domestic genomic estimates, and computational feasibility for many countries and traits. Advantages of direct multi-country genomic evaluation over GMACE are: more complete use of genomic information and more appropriate weighting of phenotypes from foreign animals. Computation was feasible for the world Brown Swiss evaluation but would require many processors and more computer memory than GMACE. Reliability gains for young bulls were large from combining genotype files, especially for the smaller populations. Genomic evaluations should benefit all breeders by improving genetic progress.

## List of abbreviations

: vector of traditional estimated breeding values; **A**: additive relationship matrix from pedigree; BV: true breeding value; EBV: estimated breeding value (traditional); c_12_: fraction of genotyped bulls common to countries 1 and 2; corr(a_1_, a_2_): genetic correlation between true BVs in countries 1 and 2; corr(e_1_, e_2_): residual correlation in countries 1 and 2; d_*i*_: ratio of genomic to total daughter equivalents in country *i*; **D**: diagonal matrix containing traditional daughter equivalents; **D**_*g*_: diagonal matrix containing daughter equivalents from genomics; **D**_*g*1_: first approximation using reliability differences; **D**_*g*2_: second approximation equating diagonals of inverses; **D**_*g*3_: third approximation setting all diagonals to the same constant; *DE*_*dau*_: daughter equivalents from domestic daughters; *DE*_*gen*_: daughter equivalents from genomics and foreign daughters; *DE*_*pa*_: daughter equivalents from parent average; *DE*_*total*_: total daughter equivalents; DYD: daughter yield deviation; : vector of genomic estimated breeding values; **G**: genomic relationship matrix; GEBV: genomic estimated breeding value; GMACE: genomic multi-trait across-country evaluation; *h*^2^: heritability; *k*: ratio of error to sire variance; MACE: multi-trait across-country evaluation; *n*: number of high reliability bulls needed to obtain 50% *REL*_*g*_; *PA*: traditional parent average; *q*: QTL effect with heavy-tailed distribution; **R**: covariance matrix among errors in y_*g*_; *REL*: traditional reliability; *REL*_*dau*_: traditional reliability from only domestic daughters; *REL*_*g*_: genomic reliability; *REL*_*pa*_: reliability of traditional parent average; **T**^-1^: inverse of genetic covariance matrix among country traits; **y**: vector of DYD or deregressed traditional evaluations; **y**_*g*_: vector of deregressed genomic evaluations; *z*: standard, normal variable; : additive genetic variance; : error variance.

## Competing interests

The authors declare that they have no competing interests.

## Authors' contributions

PV derived and programmed the multi-country evaluation of shared genotypes, simulated the Brown Swiss genomic evaluation, and drafted the manuscript. PS programmed genomic MACE. PS and PV jointly derived the formulas needed for genomic MACE and constructed the examples. Both authors read and approved the final manuscript.
